# A Sensitivity and Consistency Comparison Between Next-Generation Sequencing and Sanger Sequencing in HIV-1 Pretreatment Drug Resistance Testing

**DOI:** 10.3390/v16111713

**Published:** 2024-10-31

**Authors:** Ying Zhou, Fei Ouyang, Xiaoyan Liu, Jing Lu, Haiyang Hu, Qi Sun, Haitao Yang

**Affiliations:** 1 Department of HIV/STD Control and Prevention, Jiangsu Provincial Center for Disease Control and Prevention, Nanjing 210009, China; xyliumail@163.com (X.L.); lujing@jscdc.cn (J.L.); huhaiyang@jscdc.cn (H.H.); sq_njmu@163.com (Q.S.); yht@jscdc.cn (H.Y.); 2 Key Laboratory of Environmental Medicine Engineering of Ministry of Education, Department of Epidemiology and Health Statistics, School of Public Health, Southeast University, Nanjing 210009, China; 220213961@seu.edu.cn

**Keywords:** next-generation sequencing, HIV-1 drug resistance, consistency comparison

## Abstract

Next-generation sequencing (NGS) for HIV drug resistance (DR) testing has an increasing number of applications for the detection of low-abundance drug-resistant variants (LA-DRVs) in regard to its features as a quasi-species. However, there is less information on its detection performance in DR detection with NGS. To determine the feasibility of using NGS technology in LA-DRV detection for HIV-1 pretreatment drug resistance, 80 HIV-infected individuals who had never undergone antiretroviral therapy were subjected to both NGS and Sanger sequencing (SS) in HIV-1 drug resistance testing. The results reported in this study show that NGS exhibits higher sensitivity for drug resistance identification than SS at a 5% detection threshold. NGS showed a better consistency compared with that of SS for both protease inhibitors (PIs) and integrase inhibitors (INSTIs), with a figure amounting to more than 90%, but worse consistency in nucleotide reverse transcriptase inhibitors (NRTIs), with a consistency ranging from only 61.25% to 87.50%. The consistency of non-nucleotide reverse transcriptase inhibitors (NNRTIs) between NGS and SS was around 85%. NGS showed the highest sensitivity of 87.0% at a 5% threshold. The application of NGS technology in HIV-1 genotype resistance detection in different populations infected with HIV requires further documentation and validation.

## 1. Introduction

The HIV-1 virus, which is known to be a quasi-species, has a high replication capacity with a high error rate for reverse transcriptase and a lack of correction enzymes, leading to a high rate of nucleic acid mismatch, resulting in the production of a variety of homologous variants in infected bodies [1,2,3,4]. HIV drug resistance (HIV DR) has emerged as a major challenge for antiretroviral therapy (ART) [5]. The incredible diversity of HIV within a host and the selective pressure of HIV virions on drugs during ART make HIV resistance a complex and diverse phenomenon [6,7,8].

In China, ART is administered according to the principle of the rapid initiation of therapy, and the pre-treated monitoring of drug resistance plays a crucial role in ART regimen selection [9,10]. Sanger sequencing (SS) is conventionally used for HIV drug resistance testing and is considered the standard method for treatment-naïve individuals in this setting [11]. However, resistance variants below the 20% threshold, known as low-abundance drug-resistant variants (LA-DRVs), are not detected via SS [12]. LA-DRVs can lead to an accumulation of resistance mutations, further increasing the risk of ART failure. It has been proven that individuals carrying LA-DRVs in the HIV-1 population were correlated with treatment failure [13]. NGS technology improves the sensitivity of HIV quasi-species detection and allows quantitative identification through the high-throughput, massively parallel sequencing of individual input templates. However, many challenges exist concerning the generalized adoption of NGS for HIVDR testing, including those related to standardized analysis outputs and the selection of a threshold [14,15].

In order to clarify the efficiency of NGS detection technology in LA-DRV detection, the blood samples of HIV-1-infected individuals with no prior treatment were assessed for the detection of genotype resistance using both NGS and SS technologies by comparing the differences in the mutation rates of resistance sites in the results yielded by the two technologies to determine the feasibility of the detection method for HIV-infected individuals who had not undergone ART.

## 2. Materials and Methods

### 2.1. Sample Collection and Ethical Statement

This study was carried out using convenience sampling. Eighty HIV-1-infected individuals who were newly diagnosed and had not undergone ART were randomly selected via a sentinel survey of men who have sex with men (MSM). This study was approved by the Ethics Committee of the Jiangsu Provincial Center for Disease Prevention and Control (SL2023-B015-01).

### 2.2. Laboratory Detection

At this stage, 200 uL of plasma was extracted to obtain nucleic acid using QIAsymphony SP/AS Cellfree200_v7 (QIAGEN, Hilden, Germany) with QIAsymphony DSP Virus/Pathogen Mini Kit (QIAGEN, Hilden, Germany) nucleic acid extraction reagent. Genotype resistance detection with SS, including the amplification of protease and reverse transcriptase regions (PR-RT) and the integrase region (Int), was performed repetitively according to the cited research [16]. A Vazyme one-step PCR kit (Vazyme Biotechnology Company, Nanjing, China), the primers MAW26 (5′-TTGGAAATGTGGAAAGGAAGGAC-3′) and RT-21 (5′-CTGTATTTCTGCTATTAAGTCTTTTGATGGG-3′) for PR-RT, and the primers outF (5′-CACAYAARGGRATTGGAGGAAATG-3′) and outR (5′-TARTGGRATGTGTACTTCTGAAC-3′) for Int were used for first-round amplification. A Biotech 2*Taq PCR mix kit (Sangon Biotech Company, Shanghai, China), the primers PRO-1 (5′-CAGAGCCAACAGCCCCACCA-3′) and RT-20 (5′-CTGCCAGTTCTAGCTCTGCTTC-3′) for PR-RT, and the primers NestF (5′-AACARGTAGATAAATTAGTHAGT-3′) and NestR (5′-ATACATATGRTGYTTTACTARACT-3′) were used for target sequence amplification. The amplification products were sent to Shanghai Shenggong Company (Sangon Biotech Company, Shanghai, China) for SS using Applied Biosystems TM 3730XL (Thermo Fisher, Waltham, MA, USA). There were five sequences returned by this company for each sample. Chromaspro1.6 and MEGA11 software were used to edit contig sequences. All sequences were submitted to an HIV drug resistance database (https://hivdb.stanford.edu/, 12 January 2024) to determine the resistance level for each drug. The NGS experiment utilizes the Ion Torrent fully automated sequencing platform. The HIV pol full gene panel (Thermo Fisher, Waltham, MA, USA) is designed using HXB2 as the reference genome, targeting the entire pol gene region, spanning from 2018 to 5259 bp. The panel includes 25 amplicons and 1957 primers, with amplicon sizes ranging from 125 to 275 bp. For the experiment, the Ion Torrent™ NGS RT Kit (Thermo Fisher, Waltham, MA, USA) is used for HIV nucleic acid reverse transcription. After reverse transcription, the Ion AmpliSeq™ Kit (Thermo Fisher, Waltham, MA, USA) for Chef DL8 is employed for automated library construction, followed by template preparation and sequencing using the Ion 510™ & Ion 520™ & Ion 530™ Kit—Chef and Ion 530™ Chip Kit (Thermo Fisher, Waltham, MA, USA) [17]. The mean depth of NGS sequencing was at least 10,000×.

### 2.3. Bioinformatics Analysis

Thermo Fisher has integrated an analysis system named Ion Buffalo, with one of the tools for HIV drug resistance detection. The HIV drug resistance detection tool named HIV-holmes is one of the components of the Ion Buffalo system. The workflow of this tool is as follows: 1. Data Quality Control and Coverage Statistics. 2. Data Alignment. The data is aligned to the HXB2 reference genome using Tmap (Version 5.12). 3. Mutation Detection. Mutations and their frequencies are detected using the TVC (Version 5.12). 4. Amino Acid Annotation: Amino acid annotations are performed using the SnpEff software (version SnpEff 4.3t). 5. Sequence Assembly: Sequence assembly is conducted using IRMA under frequency models of 2%, 5%, 10% and 20% detection thresholds repetitively. 6. Drug Resistance Annotation: Mutations are annotated using the HIVDB database to obtain HIV drug-resistance mutations and their scores and resistance levels. 7. Variant Quality Control: Each assembled sequence and the mutation resulting from TVC undergo post-assembly mutation quality control (https://github.com/hivdb/hivfacts, 12 January 2024). The read data were processed with the HIV-holmes tool for drug-resistance mutations at 2%, 5%, 10%, 15% and 20% detection thresholds repetitively. The mutation counts in all the sequencing reads detected at the same site make up the detection threshold. The drug resistance sites below the 20% threshold are called low-abundance drug-resistant variants (LA-DRVs).

### 2.4. Statistical Analysis

A chi-squared test was used to analyze the statistical differences between the NGS results at a 20% or 2% threshold with the results of SS. *p* < 0.05 was considered statistically significant. McNemar’s chi-squared test was used to evaluate the consistency between the two sequencing techniques. The kappa value was used to measure the degree of consistency between the two sequencing methods. If both technologies identified the same mutation in the same individual, or if neither detected any mutations, the resistance characteristics of the two technologies would be considered consistent. Conversely, if only resistance mutations were identified via NGS or SS, the two technologies would be considered inconsistent. SS served as the reference standard. The sensitivity of NGS was defined as its ability to detect mutations identified through SS. The specificity of NGS was defined as its ability to detect mutations not detected via SS.

## 3. Results

### 3.1. The Characteristics of the Overall Rate of Pre-Treatment Drug Resistance (PDR) Mutations in 80 Samples Analyzed via SS and NGS

The NGS results showed that PDR gradually decreased with the increase in the detection threshold. At a threshold of 2%, the overall PDR rate was 25.0%, which is higher than the results obtained for other NGS thresholds in this study. The PDR rates of PI, NRTI, NNRTI and INSTI resistance were 6.3%, 5.1%, 10.3% and 6.3%, respectively, at a threshold of 2%. Except for the NNRTIs, the PDR rates of the drugs increased with the decrease in the threshold (Table 1).

Compared with the NGS results, the ability of SS to detect HIV-1 pretreatment drug resistance for PIs and NRTIs was lower, with the values for both being 2.5%. According to the results of the paired chi-squared test, when the detection threshold was 20%, there was no significant difference in the ability of NGS to detect PDR compared with that of SS (*p* = 0.884). However, when the detection threshold was reduced to 2%, the ability of NGS to identify PDR in HIV-infected individuals who had not undergone antiretroviral therapy was significantly greater than that of SS (*p* < 0.001).

**Figure 1:**
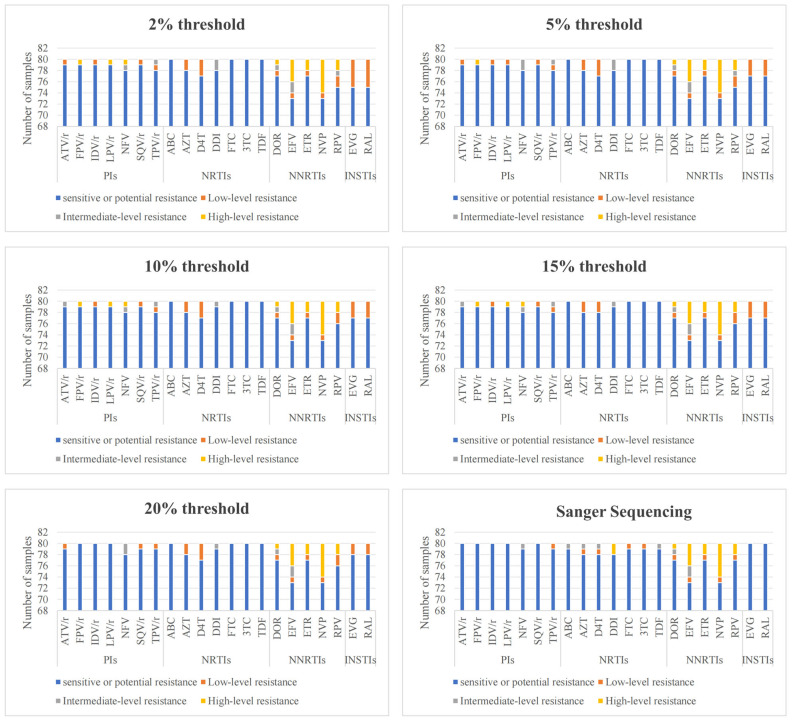
The number of ART-naïve individuals with low-level or above resistance to drugs as detected via SS and NGS.

From the results of the resistance levels with respect to the analyzed drugs, we discovered that the mutations detected by NGS against INSTIs showed a low-level resistance to first-line ART regimens (EVG and RAL) (Figure 1).

### 3.2. An Analysis of the Resistance Mutation Sites Detected via NGS at Different Thresholds

Compared to SS, NGS can detect more variant sites at lower thresholds. Fifty-two (65%) of the NGS-detected subjects had at least one resistance mutation. The number of mutations detected via NGS gradually decreased with the increase in the detection threshold. Frequencies of 90, 83, 72, 60 and 50 were detected in order of thresholds from low to high. At a 2% threshold, 32 drug-resistant samples were detected in 80 sequences, with a frequency of 90. Eleven drug-resistant samples were observed for NRTIs. The mutation frequency of S68SG (36.3%, 29/80) was the highest for NRTIs at each threshold but decreased to 10.0% (8/80) with the increase in the detection threshold of NGS. The mutation frequency of K219 was the second highest (5.0%, 4/80). There were eight mutation sites with 15 variants detected against NNRTIs. The mutation frequencies of V179E and K103KE were 10.0% (8/80) and 6.3% (5/80), respectively. The mutation frequencies for PIs and INSTIs were much lower. There were six mutation sites with six variants detected against PIs. F35L was the mutation site with the highest mutation frequency (3.8%, 3/80) for PIs. A total of five mutation sites were detected for INSTIs, and the site with the highest mutation frequency was E138EA (6.3%, 5/80) (Table 2).

### 3.3. Comparison of Consistency Between SS and NGS

**Figure 2:**
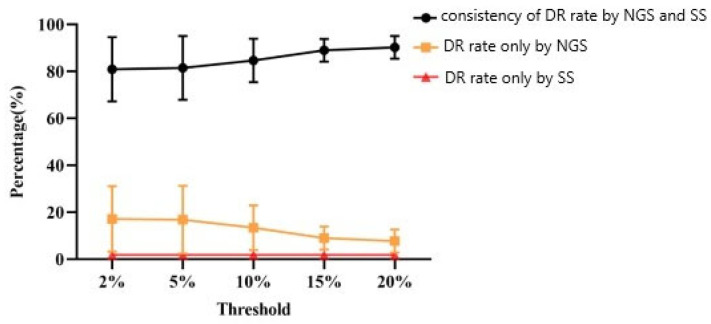
Consistency of drug resistance (DR) rates against four categories of drugs among ART-naïve individuals as identified via NGS and SS.

The consistency of the drug-resistant samples and rates detected via the two sequencing methods at different thresholds were analyzed (Table 3 and Figure 2). The consistency of the drug resistance rate against the four categories of drugs detected via NGS and SS increased with the increase in the detection threshold, while the drug resistance rate detected only via NGS decreased with the increase in the threshold. Twenty-three individuals were determined to have mutation sites via SS detection. However, 52, 51, 36, 37 and 34 individuals were determined to have mutation sites via NGS at 10%, 15% and 20% thresholds, respectively. In total, 62.50–95.00% of the results were identical between the two sequencing methods. Sensitivity and specificity analyses of SS and NGS in regard to detecting drug-resistance mutations were used to assess consistency. The consistent rates of drug-resistance mutations for PIs, NRTIs, NNRTIs and INSTIs were 92.50–95.0%, 62.50–85.00%, 78.75–87.5% and 87.50–93.75%, respectively. The majority of research subjects with NRTI-related mutations detected only via NGS had drug-resistance mutation rates of 13.75–36.25%, with the main mutation site detected being S68SG. The results of the paired chi-squared test showed that the difference between the two methods was statistically significant in terms of detecting NRTIs, NNRTIs and overall resistance mutations (Table 4). Because no samples resistant to INSTIs were identified via either NGS and SS, no INSTIs sensitivity analysis was conducted. At the 5% threshold, the sensitivity of NGS was the highest, amounting to 87.0%. The Kappa values of the two sequencing techniques for detecting arbitrary mutation sites ranged from 0.2 to 0.4, which corresponds to a general level.

## 4. Discussion

In this study, we conducted a parallel experiment consisting of genotyping drug resistance detection in HIV-1-infected ART-naïve individuals and evaluated the PDR rate at different detection thresholds by comparing the results yielded by SS and NGS. As expected, the detection rate for drug-resistance mutations was closely related to the sensitivity of NGS [18]. Specifically, NGS technology recognizes more drug-resistance mutations at lower detection thresholds with higher sensitivity. It provides more comprehensive resistance information than SS technology.

In this study, the overall PDR rate resulting from NGS testing was 25%, which is higher than that for SS. The drug resistance rates for other drug categories were also higher. Using NGS in a similar fashion, Lataillade et al. reported that 30.5% (43/141) of study subjects from five continents (Africa, Asia, Europe and North and South America) were determined to have drug-resistance mutations before treatment at a >1% threshold [19]. In fact, our PDR is quite close to the rate reported by Lataillade, considering the 2% threshold we chose. In another study, no major INSTIs-related mutations were identified among ART-naïve individuals [20,21]. In our study, NGS showed obvious superiority in detecting mutation sites against PIs and INSTIs. M46I, I47AV, F53L and Q58E were detected via NGS, all of which are mutations that can induce low-level or above resistance to PIs. However, in this study, only one M46I and one Q58E were determined via SS. E138EA, detected via NGS, can induce low-level or above resistance to INSTIs, and it was not detected via SS. It is worth noticing that in this study, NGS did not show an additional advantage in detecting the mutations of NNRTIs. Mutations against NRTI or NNRTIs, detected via NGS but not SS, cannot affect viral resistance. Previous studies showed that M46L, K103N, V179D and M184V are the mutations most frequently and easily detected via SS in ART-naïve individuals [22]. Unfortunately, the mutation sites of K103N and V179E were not detected via NGS, but they were detected via SS in two individuals, respectively. Although NGS is more sensitive in theory, the lack of detection ability for K103N and V179E makes us doubt the accuracy of SS in these two individuals since nucleotide misincorporation and PCR-mediated recombination more easily occur in long fragment sequencing like SS. K103N is an important drug resistance site possessing clinical significance. It can decrease sensitivity to NVP and EFV. It is suggested that some clinical indexes like viral load or CD4 counts can be considered to estimate the existence of drug mutations. The methodology of NGS is based on the amplification, sequencing and splicing of small fragments. When there are short fragments, there will be a certain error tolerance rate. The accuracy of IonS5 is almost 98%, as reported in [23]. The mutations not found were E44EDV at a 2% threshold, and T66A at 2% and 5% thresholds. The accuracy of IonS5 is acceptable, amounting to 97.88% at a 2% threshold and 98.8% at a 5% threshold. However, the quality control of NGS for HIV-1-genotype-resistance testing is still a challenge and needs to be addressed [24].

This study reveals significant differences between SS and NGS for the detection of drug-resistant mutations and drug resistance levels. With these differences becoming more pronounced as the threshold level decreases, NGS offers a sharp increase in the detected mutation sites below the 5% threshold. By comparing the consistency of these two sequencing methods for discovering the mutation sites and the number of drug-resistance mutations in all subjects, we found that there was high consistency between the two methods for the identification of drug-resistance mutations associated with PIs and INSTIs, with the corresponding value reaching more than 90%. However, the consistency for drug-resistance mutations associated with NRTIs was relatively low, ranging from 61.25% to 87.50%. In addition, the proportion of individuals with NRTI-drug-related mutations identified only via NGS increased gradually with the decrease in the detection threshold, and the proportion of NRTI-drug-related mutations identified via NGS was higher than that of other categories of drug-related mutations, suggesting that resistance mutations associated with NRTIs are more easily detected via NGS, albeit with poor accuracy. Although the consistency between the resistance profiles of SS and NGS in this study is lower than that reported in previous studies (83–92%) [25], this may be because we selected pre-treatment HIV-1 subjects. For those who have received antiviral therapy, under the conditions induced by drugs, drug-resistance mutations are induced and maintain survival in vivo. These mutation sites are more easily detected using SS and NGS. From the virus itself, without the stress caused by drugs, the mutation of HIV occurs more randomly in vivo because of its features as a quasi-species. This kind of variability exists in different frequencies that make their detection dependent on different sensitive methods. From the host, signature apolipoprotein B mRNA editing enzyme catalytic polypeptide-like (APOBEC)-mediated G-to-A hypermutation in a sample at different detection thresholds allowed for the determination of the optimal LA-DRVs [26]. These mutations may not be stable or have no relationship with drug resistance. However, they can affect the accuracy of test results, especially for highly sensitive methods like NGS. But no APOBEC-related mutations were included in this study. Both virus variation and APOBEC-related mutation could be the cause for consistency in mutation frequencies. So, an undesired consistency between the two methods for genotype drug resistance testing in HIV-infected individuals who had not been subjected to ART was observed. Although there are differences between different studies, it is already recognized that NGS has higher sensitivity than SS, and we proved this in our study. If the efficacy of NGS in different populations and at different thresholds can be determined; then, the NGS results applied to HIV-1 genotype resistance detection will be more reliable and analyzable.

## 5. Conclusions

Compared to Sanger sequencing, the sensitivity of NGS has been proven to be higher. NGS is viable for HIV-1 genotype resistance detection in HIV-infected ART-naïve patients for PIs and INSTIs. It is crucial to determine which one is a better predictor of treatment responses since NGS and SS are less consistent in regard to NRTIs and NNRTIs. Furthermore, the application of NGS technology in HIV-1 genotype resistance detection in different populations infected with HIV requires further documentation and validation.

## Figures and Tables

**Table 1:** The overall PDR rate based on NGS and SS.

Resistance Patterns	Sanger Sequencing	Next-Generation Sequencing				
		20% Threshold	15% Threshold	10% Threshold	5% Threshold	2% Threshold
Resistant samples, *n* (%)	11 (13.8)	14 (17.9)	16 (20.5)	17 (21.8)	19 (23.8)	20 (25.0)
PI resistance, *n* (%)	2 (2.5)	3 (3.8)	5 (6.3)	5 (6.3)	5 (6.3)	5 (6.3)
NRTI resistance, *n* (%)	2 (2.5)	2 (2.6)	2 (2.6)	3 (3.8)	4 (5.1)	4 (5.1)
NNRTI class resistance, *n* (%)	8 (10.0)	8 (10.3)	8 (10.3)	8 (10.3)	8 (10.3)	8 (10.3)
INSTI resistance, *n* (%)	0	2 (2.6)	3 (3.8)	3 (300.8)	3 (3.8)	5 (6.3)

PIs, NRTIs, NNRTIs and INSTIs: all drugs targeting HIV-1 protease, nucleotide reverse transcriptase, non-nucleotide reverse transcriptase and integrase provided by the HIV drug resistance database (Stanford University). *n*: number of patients with mutations.

**Table 2:** Resistance mutation statistics at different thresholds detected via SS and NGS.

Drug Classes	Mutation Site	Next-Generation-Sequencing Detection Threshold					Sanger Sequencing
		2%	5%	10%	15%	20%	
PIs	L33F	3	3	3	3	3	3
	M46I	1	1	1	1	1	1
	I47AV	1	1	1	1	0	0
	F53L	3	3	3	3	1	0
	Q58E	1	1	1	1	1	1
	L89LMV	1	1	1	1	1	0
	total	11	10	11	11	9	5
NRTIs	E40F	0	1	1	1	1	0
	M41L	1	1	1	1	1	1
	E44EDV	0	1	0	0	0	0
	S68SG	27	27	20	12	8	0
	S68G	2	2	2	2	2	3
	D67N	1	1	1	1	1	1
	T69D	1	1	1	1	1	1
	D67del	0	0	0	0	0	1
	T69TADN	1	1	0	0	0	0
	F77FL	1	0	0	0	0	0
	T215TS	1	1	1	0	0	0
	K219KN	2	2	2	2	1	0
	K219KQ	1	1	0	0	0	0
	K219KR	1	1	0	0	0	0
	total	39	40	29	20	15	7
NNRTIs	K101E	1	1	1	1	1	1
	K103KE	5	5	5	2	1	0
	K103N	2	2	3	3	3	4
	K103NS	1	1	0	0	0	0
	K103S	1	1	1	1	1	1
	V106VI	1	0	0	0	0	0
	E138EA	1	1	1	1	1	0
	E138EG	1	1	1	1	1	1
	E138EK	1	1	0	0	0	0
	V179D	3	3	3	3	3	1
	V179E	8	8	8	8	7	5
	Y181C	1	1	1	1	1	1
	Y181V	1	1	1	1	1	1
	G190S	1	1	1	1	1	1
	P225PH	1	1	1	1	1	1
	total	29	28	27	24	22	17
INSTIs	T66A	0	0	1	0	0	0
	E138EA	5	3	3	3	2	0
	A128AT	1	0	0	0	0	1
	S153A	4	1	1	1	1	1
	D232N	1	1	1	1	1	0
	total	11	5	5	5	4	2

**Table 3:** Consistency of the drug-resistant (DR) samples identified via NGS and SS.

Detection Threshold	Drug-Resistant Samples	PIs	NRTIs	NNRTIs	INSTIs
2%	Only detected via NGS	6.25%	36.25%	18.75%	11.25%
	Only detected via SS	1.25%	1.25%	2.50%	1.25%
	Consistency	92.50%	62.50%	78.75%	87.50%
5%	Only detected via NGS	6.25%	37.50%	16.25%	7.50%
	Only detected via SS	1.25%	1.25%	2.50%	2.50%
	Consistency	92.50%	62.50%	81.25%	90.00%
10%	Only detected via NGS	6.25%	26.25%	15.00%	6.25%
	Only detected via SS	1.25%	1.25%	2.50%	2.50%
	Consistency	92.50%	72.5%	82.50%	91.25%
15%	Only detected via NGS	5.00%	15.00%	11.25%	5.00%
	Only detected via SS	1.25%	1.25%	2.50%	2.50%
	Consistency	93.75%	83.75%	86.25%	92.50%
20%	Only detected via NGS	3.75%	13.75%	10.00%	3.75%
	Only detected via SS	1.25%	1.25%	2.50%	1.25%
	Consistency	95.00%	85.00%	87.50%	95.00%

**Table 4:** Sensitivity and specificity comparison between NGS and SS in regard to DR testing among ART-naïve individuals.

	NGS	SS		Sensitivity	Specificity	*p*	Kappa Value	95% CI
		Detected	Not Detected					
PIs								
2%/5%/10%	Detected	4	5	80.0%	93.3%	0.109	0.534	0.211–0.857
	Not detected	1	70					
15%	Detected	4	4	80.0%	94.6%	0.109	0.583	0.257–0.810
	Not detected	1	71					
20%	Detected	4	3	80.0%	96.0%	0.109	0.640	0.315–0.965
	Not detected	1	72					
NRTIs								
2%	Detected	3	29	75.0%	61.8%	<0.001	0.085	−0.042–0.212
	Not detected	1	47					
5%	Detected	3	30	75.0%	60.5%	<0.001	0.080	−0.042–0.202
	Not detected	1	46					
10%	Detected	3	21	75.0%	72.4%	<0.001	0.141	−0.033–0.315
	Not detected	1	55					
15%	Detected	3	12	75.0%	84.2%	0.003	0.257	−0.004–0.518
	Not detected	1	64					
20%	Detected	2	11	50.0%	86.8%	0.006	0.278	0.004–0.552
	Not detected	1	66					
NNRTIs								
2%	Detected	11	10	84.6%	85.1%	0.019	0.558	0.342–0.774
	Not detected	2	57					
5%	Detected	11	8	84.6%	88.1%	<0.001	0.409	0.168–0.650
	Not detected	2	59					
10%	Detected	11	9	84.6%	86.6%	0.033	0.585	0.369–0.801
	Not detected	2	58					
15%	Detected	11	8	84.6%	88.1%	0.055	0.613	0.399–0.823
	Not detected	2	59					
20%	Detected	11	7	84.6%	89.6%	<0.001	0.599	0.322–0.796
	Not detected	2	60					
mutation variant								
2%	Detected	20	32	87.0%	43.9%	<0.001	0.224	0.071–0.377
	Not detected	3	25					
5%	Detected	20	30	87.0%	47.4%	<0.001	0.254	0.095–0.413
	Not detected	3	27					
10%	Detected	19	25	82.6%	56.1%	<0.001	0.305	0.129–0.481
	Not detected	4	32					
15%	Detected	19	18	82.6%	68.4%	<0.001	0.417	0.231–0.603
	Not detected	4	39					
20%	Detected	17	19	81.0%	67.8%	<0.001	0.396	0.206–0.586
	Not detected	4	40					

## Data Availability

Data are contained within this article.
